# Hystricognathy *vs* Sciurognathy in the Rodent Jaw: A New Morphometric Assessment of Hystricognathy Applied to the Living Fossil *Laonastes* (Diatomyidae)

**DOI:** 10.1371/journal.pone.0018698

**Published:** 2011-04-07

**Authors:** Lionel Hautier, Renaud Lebrun, Soonchan Saksiri, Jacques Michaux, Monique Vianey-Liaud, Laurent Marivaux

**Affiliations:** 1 Museum of Zoology, University of Cambridge, Cambridge, United Kingdom; 2 Anthropologisches Institut und Museum, Universität Zürich, Zürich, Suisse; 3 Laboratoire de Paléontologie, Institut des Sciences de l'Evolution de Montpellier, UMR-CNRS 5554, Cc 064, Université de Montpellier 2, place Eugène Bataillon, Montpellier, France; 4 Department of Biology, Faculty of Science, Mahasarakham University, Tambon Khamriang Kantarawichai District, Mahasarakham, Thailand; University College London, United Kingdom

## Abstract

While exceptional for an intense diversification of lineages, the evolutionary history of the order Rodentia comprises only a limited number of morphological morphotypes for the mandible. This situation could partly explain the intense debates about the taxonomic position of the latest described member of this clade, the Laotian rock rat *Laonastes aenigmamus* (Diatomyidae). This discovery has re-launched the debate on the definition of the Hystricognathi suborder identified using the angle of the jaw relative to the plane of the incisors. Our study aims to end this ambiguity. For clarity, it became necessary to revisit the entire morphological diversity of the mandible in extant and extinct rodents. However, current and past rodent diversity brings out the limitations of the qualitative descriptive approach and highlights the need for a quantitative approach. Here, we present the first descriptive comparison of the masticatory apparatus within the Ctenohystrica clade, in combining classic comparative anatomy with morphometrical methods. First, we quantified the shape of the mandible in rodents using 3D landmarks. Then, the analysis of osteological features was compared to myological features in order to understand the biomechanical origin of this morphological diversity. Among the morphological variation observed, the mandible of *Laonastes aenigmamus* displays an intermediate association of features that could be considered neither as sciurognathous nor as hystricognathous.

## Introduction

The mammalian masticatory apparatus is a highly plastic region of the skull, which explains why the associated features are used as diagnostic phylogenetic attributes. Among mammals, the radiation of rodents constitutes a special case. Rodents are considered to be one of the great successful groups in the evolutionary history of mammals, and few mammal clades have been studied as extensively as the Order Rodentia. The modern representatives of the Order, around 2200 species [1], are spread across every continent barring Antarctica. They constitute roughly half of the current mammalian diversity. This astonishing specific diversity is shown most notably in terms of ecology as they occupy the majority of the ecosystems on the planet. Moreover, rodents show one of the most extreme differentiation of the masticatory apparatus with a single pair of upper and lower incisors highly specialized for gnawing, and a small number of cheek teeth for chewing in association with the development of antero-posterior movements [2]. As such, the masticatory apparatus was early recognized and used as diagnostic phylogenetical attribute. However, many studies showed that the arrangements of masticatory muscles could not be used to classify rodents at the suborder level [3], [4], [5], [6].

**Figure 1:**
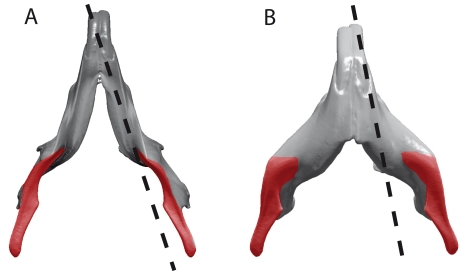
Mandibular types defined by Tullberg [7] in ventral view. A, sciurognathous jaw; B, hystricognathous jaw. The angular process is coloured in red.

While exceptional for an intense diversification of lineages, the evolutionary history of the order Rodentia retains only a small number of morphological solutions for the skull and mandible [4], [5]. Such a situation could be partly due to strong functional constraints that affected mastication, thereby limiting the number of possible pathways and promoting convergent evolution as a result. Considering the relative position of the angular process relative to the plane of the incisors, Rodentia were commonly divided into two suborders: Sciurognathi and Hystricognathi [7], [8], [9] (Fig. 1). The sciurognathous jaws are characterized by an angular process originating in the same plane that includes the alveolus of the incisors. By contrast, the hystricognathous jaw shows the origin of the angular process distinctly lateral to the plane of the alveolus of incisors. Hereafter, Sciurognathi and Hystricognathi (sciurognaths and hystricognaths as synonym) will be used to qualify the two suborders defined by Tullberg [7], whereas sciurognathy and hystricognathy (sciurognathous and hystricognathous as adjectives) will refer to the condition of the mandible that could be developed in one or the other suborders.

**Figure 2:**
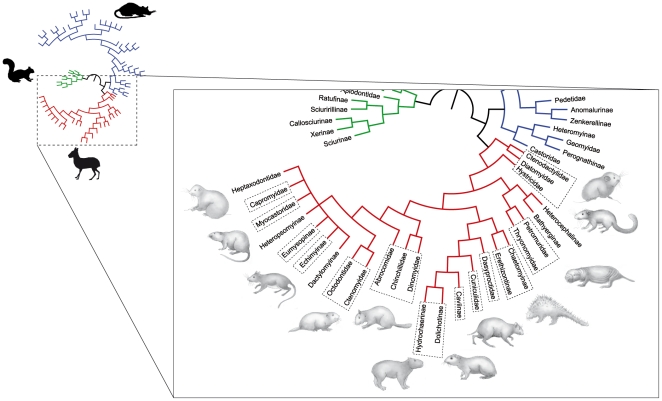
A phylogenetic tree of the rodent clade Ctenohystrica derived from molecular analyses [13], [66]. Note the position of the clade ctenodactylids*-Laonastes* as the sister group of Hystricognathi. Red, Ctenohystrica; blue, mouse relative clade; green, sciurid relative clade. Dashed lines highlight the sample composition. Original artwork by Laurence Meslin, © Laurence Meslin – CNRS.

The discovery of the Laotian rock rat *Laonastes aenigmamus* [10] recently revived the debate around the hystricognathy by underlining the ambiguity on its definition. *L. aenigmamus* has first been considered as the sole member of a new hystricognathous family Laonastidae. However, a re-examination of the specimens [11] has shown that this species could represent a surviving member of the extinct family Diatomyidae among the “ctenodactyloid rodents”, *i.e.* a sciurognathous family [12]. More recently, molecular analyses [13] unambiguously confirmed the paleontological view in demonstrating that *L. aenigmamus* is the sister group of Ctenodactylidae (within the monophyletic Ctenohystrica – Fig. 2). On the one hand, such a discovery of a new species offered a rare opportunity to study an original osteological and myological combination among Ctenohystrica [14]. On the other hand, the debate around the hystricognathous condition of its mandible showed the necessity of revisiting the entire morphological diversity of the mandible of extant and extinct hystricognathous rodents in order to better understand the evolution of this conservative morphological feature.

The rise of molecular phylogeny methods has allowed an independent evaluation of the affiliation between living species. However, only the phylogenetic methods that depend on the analysis of anatomical characteristics can take both fossil and modern species into account, and still remain applicable to the entire order Rodentia. The fossil record is to the understanding of the process of evolution what the “Rosetta stone” was to the understanding of hieroglyphics: the key to interpreting the evolution of forms. For all this, most of the information at our disposal in studying the fossil material, with the exception of its geological age, pertains to its morphology. Thus the means to quantify the morphology has become of great importance. In parallel with progresses in molecular genetics, the advent of geometric morphometric methods marked a milestone in quantitative phenotypic analysis [15], [16], [17], allowing for finer interpretations of the fossil record.

With the large set of morphological and molecular data available, reinterpretation of the fossil record within a molecular based phylogenetic framework becomes possible. Here, we propose a new method to recognize the hystricognathous condition of a lower jaw that we apply to the mandible of the living fossil *Laonastes aenigmamus*. The aim of this study is not to perform a phylogenetical analysis or to find new informative phylogenetic characters, but is to use information about phylogeny and ecology to assess evolutionary processes that could explain a morphological differentiation of hystricognathous jaws. This work leads to a redefinition of hystricognathy and has implications for the interpretation of the fossil record of early hystricognaths.

## Materials and Methods

### Sample composition

**Figure 3:**
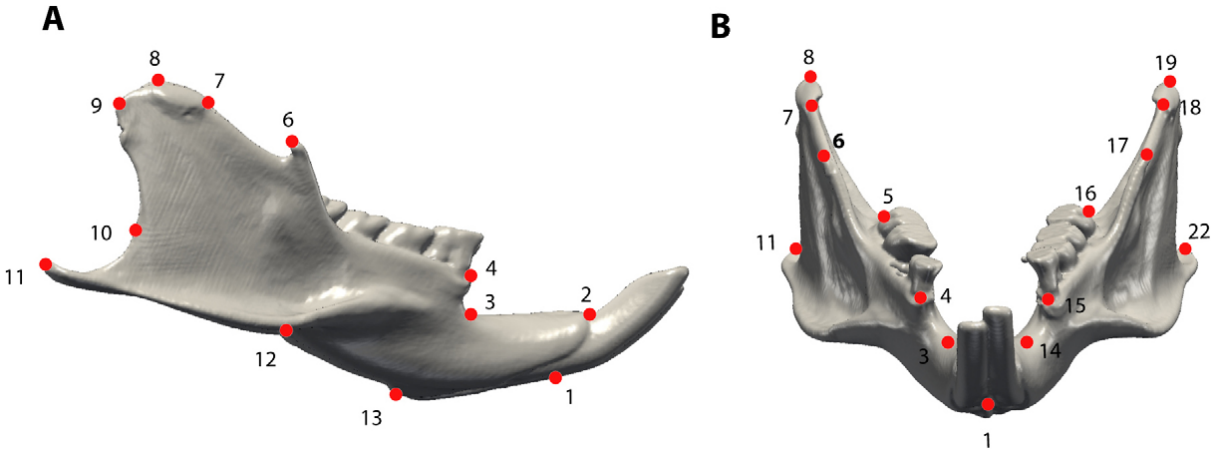
Landmarks digitized on the mandible. A, lateral view; B, anterior view.

The Ctenohystrica (*sensu* Huchon et al. [18]: Ctenodactylidae+Diatomyidae and Hystricognathi) exemplify a rich evolutionary history in the Old and New World. As a clade, they have the essential assets to fulfil the objectives set here: they are very diversified, with a wide range of ecomorphological adaptations and they include both sciurognathous (Ctenodactylidae) and hystricognathous members (Hystricognathi). The material studied comes from the collection of the *Museum National d’Histoire Naturelle* in Paris (MNHN, collection *Vertébrés supérieurs Mammifères et Oiseaux*), the Natural History Museum in London (BMNH), the Mahasarakham University Herbarium (MSUT), and of the *Institut des Sciences de l’Evolution de Montpellier 2* (ISE-M). We analysed 177 mandibles belonging to sciurognathous and hystricognathous rodents of both sexes, representing 43 genera and 16 families (Fig. 2): Abrocomiidae, Capromyidae, Cuniculidae, Caviidae, Chinchillidae, Ctenodactylidae, Ctenomyidae, Dasyproctidae, Diatomyidae, Dinomyidae, Echimyidae, Erethizontidae, Hystricidae, Octodontidae, Petromuridae and Thryonomyidae (see list in Appendix S1). Members of the Bathyergidae, which are fossorial rodents, could not be considered in this study because the morphology of their mandible is too divergent (highly specialized) to allow a clear recognition of certain landmarks (e.g. landmarks 3, 12, 13, 14 and 23; see analysis protocol Fig. 3). For this study, three specimens of Laotian Rock rats, *Laonastes aenigmamus*, were collected in 2007 in the Khammouan Province of the Lao People's Democratic Republic (Lao PDR). All the specimens were captured by villagers from Mauang Village (Thakhek district) and collected in the Thakhek market (Thakhek district) in 2007 and are now deposited at the Mahasarakham University Herbarium (University of Mahasarakham, Tambon Khamriang Kantarawichai district, Thailand).

### Geometric morphometric methods

The mandibular form was quantified with 23 anatomical landmarks distributed approximately equally over the mandible (Fig. 2). Digital volume data of all specimens were acquired using a Microscribe 3-D digitizer and using X-ray micro-computed tomography (µCT). Because the mandible of rodents is constituted by a unique dentary bone of relatively simple shape, most of the landmarks taken were of type 2 (i.e. maxima of curvature – Fig. 3; [15]). Each individual was digitized twice in order to assess measurement error. All configurations (set of landmarks) were superimposed following the Procrustes method of generalized least squares superimposition (GLS scaled, translated, and rotated configurations so that the intralandmark distances were minimized) following the method used by Rohlf [19] and Bookstein [15]. Subsequently, mandibular form of each specimen was represented by its centroid size S, and by its multidimensional shape vector v in linearized Procrustes shape space. Shape variability of the mandible was analysed by principal components analysis (PCA) of shape [16]. Analysis and visualization of patterns of shape variation were performed with the interactive software package MORPHOTOOLS [20], [21], [22], [23]. This program is still under development and some functionalities (not those used in use publication) still need to be tested. A public version is currently being developed (contact renaud.lebrun@univ-montp2.fr for further information). In order to take into account the potentially confounding effects of size allometry on shape, size-corrected shapes were obtained as follows. Regressions of Procrustes coordinates against the logarithm of centroid size were computed for all families (except for all mono-specific families), yielding family-specific allometric shape vectors (*ASVf*). The *ASVf* represent directions in shape space which characterize family-specific allometric patterns of shape variation. A common allometric shape vector (*ASVc*), obtained as the mean of all the *ASVf*, provided a direction in shape space that minimizes potential divergence in mandibular allometric patterns across families (see [23] and [24] for further details concerning this methodology). *ASVc* was then used to decompose the shape of each species-wise mean shape and of each family-wise mean shape into size-related (*vs*) and size independent (*vi*) components.

The South American hystricognathous rodents (i.e. the Caviomorpha) are remarkable among Ctenohystrica in showing several examples of parallel evolution. For instance, the differentiation in diet and habitat has occurred independently in two monophyletic groups, the Cavioidea [25] and the Octodontoidea [26]. This parallel evolution gave us a unique opportunity to separate the effect of phylogenetic and ecological constraints on morphological evolution. Manovas and Canonical Variate Analyses were performed on the Principal Component scores of each species-wise mandibular mean shapes (*vi*) in order to assess the effects of different factors on mandibular shape variation; clades (families), diet and type of habitat [27], [28]. Following Towsend and Croft [28], five categories of diets were considered: omnivorous, fruit-leaf, fruit-seed, grass, and roots. Four types of habitats were set apart: open areas, woody areas, burrowers, and ubiquists [27]. The terms “type of habitat” and “diet” refer to the usual habitat and principal diet and are given in the appendix. In order to quantify mandibular shape affinities at the family level, family-wise mean mandibular shapes were clustered using the UPGMA (unweighted pair-group method) on original shape data and shape data corrected for allometry. The UPGMA trees were computed using Phylip [29].

## Results

### Morphological variation of the mandibles among Ctenohystrica

**Figure 4:**
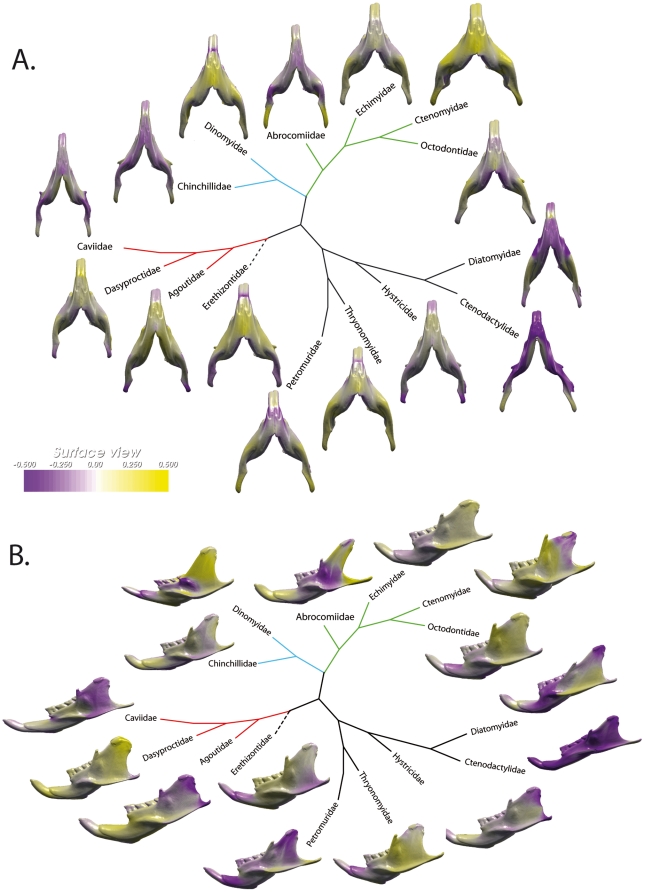
Morphological variation of the mandible among Ctenohystrica. A, ventral view; B, lateral view. Colors indicate the relative amount of change in local area that was necessary to attain that shape, with the reference being the consensus shape. Yellow and violet code for an increase and decrease in surface area, respectively, and white indicates isometry. Scale unit: local area/same local area of the reference shape. On a ventral view, the yellow color will code for the lateralization of the angular process and thus hystricognathy whereas violet will characterize the sciurognathous condition.

We observe an important morphological variation of the mandible within the Ctenohystrica (Fig. 4). The sciurognathous members (i.e. Ctenodactylidae) are well discriminated. A differentiation is also well expressed at a super-familial level as both Cavioidea [25] and Octodontoidea members [26] occupied variable positions in the morphospace of the mandibles. Two extreme morphotypes can be recognized. The “octodontoid” type [30], displayed by Ctenomyidae, is characterized by a short diastema, a short incisivo-condylar length, a high and rounded mandibular condyle well individualized, parallel tooth rows, and a narrow angular process distinctly lateral to the plan defined by the alveolus of the incisors. This “octodontoid” type is clearly distinct from the “cavioid” type (seen in Caviidae - [30]) that displays a long diastema, a long incisivo-condylar length, a large and low condylar process weakly individualized, convergent tooth rows, and an angular process, which is positioned distally and slightly laterally relative to the plane of the incisors. Among this variation, the morphology of the mandible of *Laonastes aenigmamus* is unique in displaying a short diastema, a low condyle, parallel tooth rows, and an angular process, which is slightly lateral to the plane of the alveolus of the incisors.

**Figure 5:**
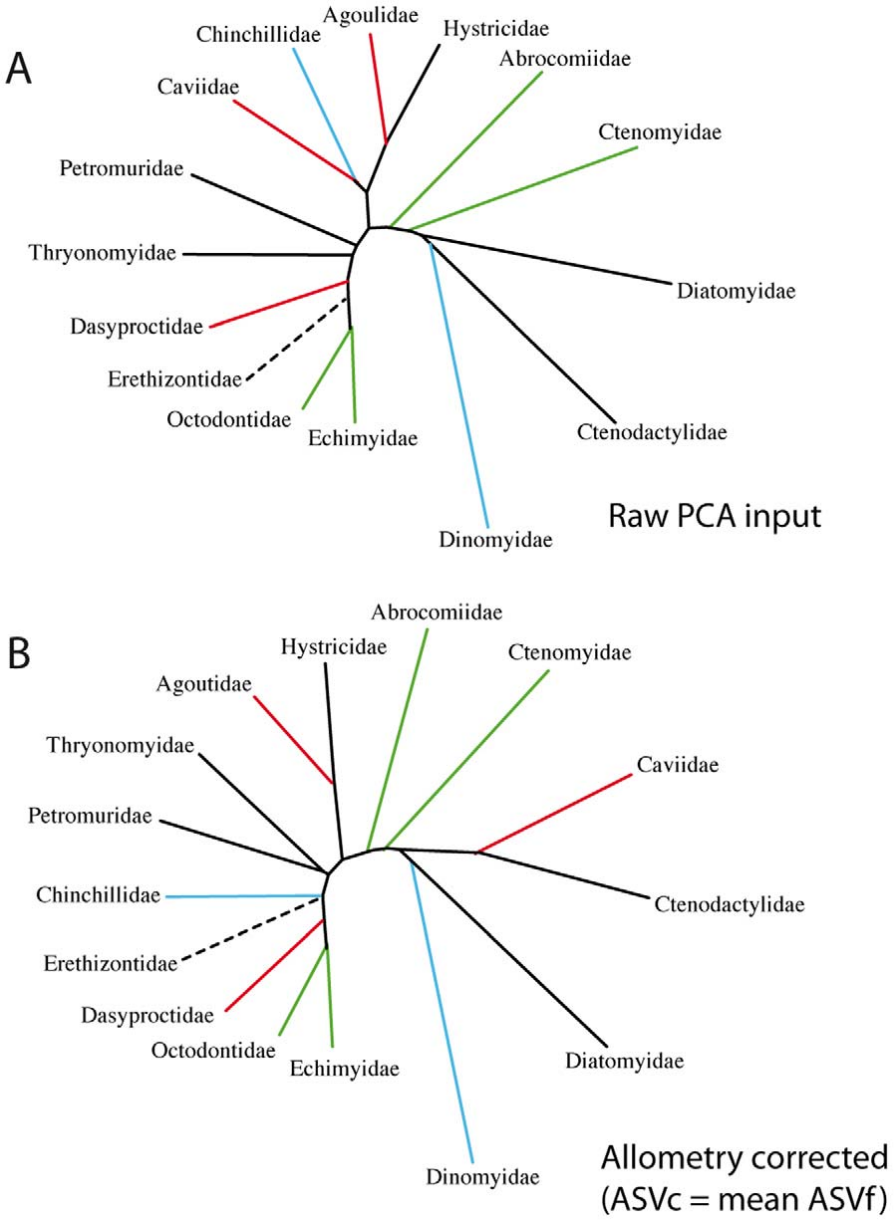
Phenetic trees based on mandible shape. A, tree reflecting simple morphological affinities between Ctenohystrica families; B, size-corrected mandibular shape. Note the position of Caviidae close to Ctenodactylidae (i.e. sciurognathous rodents) in the second tree.

On the ventral view (Fig. 4A), the lateralization of the mandible, i.e. the key feature used to define hystricognathy, appear to be very variable among hystricognathous jaws. This lateralization is particularly weak in the families Caviidae, Chinchillidae, and Hystricidae (Fig. 4A). We observe an extreme case of low lateralization of the angular process in the caviid genus *Kerodon*, which displays an angular process in the same plane that includes the alveolus of the incisors. The UPGMA tree (Fig. 5A) reflects the whole mandible morphological affinities. The morphological variation of the mandibles is highly incongruent with the well-supported phylogeny taken as reference (Fig. 2). The Diatomyidae are strongly associated with the sciurognathous family (Ctenodacylidae). The extreme reduction of the coronoid process in Dinomyidae could explain their location close to the ctenodactylids.

### Morphological variation and allometry

Allometry is a well-known factor, which is thought to intervene in the evolution of morphological features, especially in rodents [31]. The multivariate regression of the shape component on size, estimated by the logarithm of the centroid size, was highly significant (F = 16.5, p <0.001, dl = 105). With such condition, allometry is therefore expected to explain a substantial part of shape variation and to play an important role for determining the pattern of morphological diversification of the mandible. Size-corrected mandibular phenetic affinities are described on Fig. 5B, this UPGMA tree appears quite different from the previous one reflecting mandible morphological affinities only (Fig. 5A). In correcting for evolutionary allometry, mandible evidence places the Caviidae close to the sciurognathous Ctenodactylidae.

### Morphological variation and adaptation

**Figure 6:**
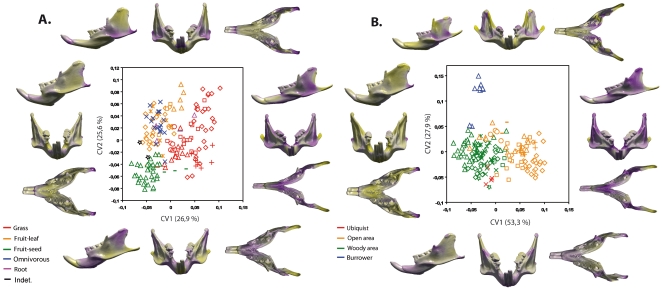
Canonical variate analyses and associate patterns of morphological transformation for the mandible. A, diet; B, habitat. Symbols indicate different clades: open stars, Diatomyidae; bars; Petromuridae; open circles, Thryonomyidae; crosses, Hystricidae; open triangles, Octodontoidea; open diamonds, Cavioidea; open squares, Chinchilloidea; trifid crosses, Erethizontoidea; “plus” symbol, Ctenodactylidae. Yellow and violet colors of the osteological features, same legend as Figure 4.

Manovas indicate a significant morphological differentiation of the mandible between rodents of different diet (F = 3.09, p<0.001,dl = 5). Morphological groups reflecting distinct types of diet are displayed along the first discriminant axis (Fig. 6A). This axis mainly discriminates grass eaters from other types of diet by separating robust mandibles with a strong symphysis, short parallel tooth rows, a thin angular process, and a condyle distally positioned, from mandibles showing a slender symphysis, elongated and convergent tooth rows, a distally positioned angular process, and a condyle anteriorly positioned. In terms of shape variation, the second discriminant axis separates mandibles that show an elongated angular process and a low condyle relative to the alveolar plane, from mandibles having a reduced angular process associated with a higher position of the condyle. This axis allows discriminating rodents that eat fruit and seeds.

Mandibular shapes in relation to the type of habitat (Fig. 6B) can be completely discriminated (F = 1.51, p<0.001, dl = 3). The first discriminant axis separates mandibles with a high horizontal ramus, a robust ascending ramus, a wide condyle, and a reduced angular process, from mandibles characterized by a low horizontal ramus, a slight ascending ramus, a narrow condyle, and an angular process that appears distal in position. This axis allows distinguishing rodents living in open and woody areas. The second discriminant axis mainly separates mandibles having spaced tooth rows, and reduced angular and coronoid processes, from mandibles showing close tooth rows, and angular processes distally positioned and highly divergent. This axis discriminates the burrowers from other rodents.

## Discussion

### Hystricognathy *vs* sciurognathy

Our morphological data set, associated with the great amount of phylogenetic results, allows an assessment of the morphological variation in mandibles among both hystricognaths and sciurognaths. By quantifying the blueprints of the morphological variation of hystricognathous mandibles, we demonstrated that the term “hystricognathy” is not shown to cover a unique mandibular morphology (Fig. 4A). In relation to their environment and/or their diet, we showed that the morphological variation of the mandible is great within the current shapes of hystricognathous jaws. Confirming previous results [30], two extreme morphotypes (cavioid and octodontoid) were recognized among mandibles of Hystricognathi. As such, Vassalo and Verzi [30] recognized them as “slight” and “strong” hystricognathous condition. In fact, these morphotypes reflect the morpho-anatomical differences first identified by Tullberg [7] for establishing his long-standing classification based on the orientation of the angular process. Moreover, a continuity of morphologies exists between these two extreme morphotypes (Fig. 4), but all of these morphological combinations are recognized as hystricognathous jaws even if some of them (e.g. Caviidae or Chinchillidae) appear to be more similar to “true” sciurognathous jaws than hystricognathous ones. Considering the fossil record [32], the weak lateralization of the angular process of the mandibles of some extant members of the Caviidae could clearly be considered as examples of evolutionary reversals.

Current mammal diversity is the result of multiple radiations linked to the invasion of new ecological niches. The various groups of hystricognaths developed a wide trophic range shown first and foremost by a significant morphological differentiation of their masticatory apparatus. We found a significant morphological differentiation of the mandible among hystricognathous rodents that are characterized by distinct diet or habitat (Fig. 6). Mandibles of the “octodontoid” type characterized rodents living in woody areas and eating both fruits and seeds. The morphological features developed by members of the “cavioid” type are very similar to those found in rodents living in open habitat and/or in grass eaters. The morphology of the mandible of the “cavioid” type appears also highly related to the acquisition of hypsodont cheek teeth. The same association of features was observed in the extinct family Theridomyidae (genus *Issiodoromys* [33]). Studying this morphological differentiation thus requires a precise knowledge of the masticatory mechanics.

### Biomechanics of hystricognathous jaw

The movements of the mandible associated with feeding are performed by the masticatory muscles and are a function of the dental morphology [34], [35], the anatomy of the masticatory apparatus, and the shape and position of the mandibular joint [36], [37]. Mechanical advantages were performed by changing the origin position of the masseteric musculature [33]. Many modifications of the arrangement of the masseteric complex have occurred in the evolutionary history of hystricognaths. The evolutionary transitions between different types of chewing modes should be explained by moderate morpho-functional modifications constraint by the necessity of preservation of efficient occlusion [33], [35].

**Figure 7:**
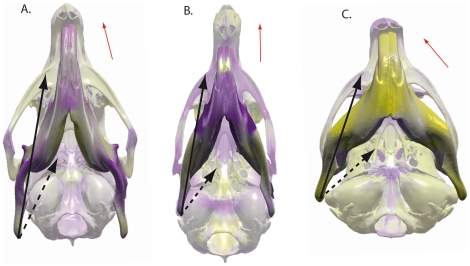
Ventral view of the skull and the mandible in rodents of the “Cavioid” type (A), in *Laonastes* (B), and in rodents of the octodontoid type (C). Black arrows show the origin and insertion of the superficial portion of the masseter muscle. Dashed arrows represent the internal pterygoid muscle. Red arrows express the direction of mastication. Yellow and violet colors of the osteological features, same legend as Figure 4.

Most of the caviomorph rodents are characterized by an oblique mastication associated with flattening of the molar occlusal surface [30]. The morphological differentiation observed among the mandible of hystricognathous rodents could be linked to the mechanics of their whole masticatory apparatus. The most important difference concerns the position of the mandibular condyle (Fig. 4B). This study only focused on the masseter and internal pterygoid muscles because they represent the greater part of the jaw elevating musculature. The temporal and external pterygoid muscles have not been considered here but these muscles probably have an important role for the stabilization of the mandible during the chewing stroke [38]. The octodontoid type shows a high mandibular condyle and an important latero-medial orientation of the internal pterygoid muscles in association with oblique chewing movements ([30] – Fig. 7C). Conversely, the mandible of cavioid type is characterized by a low mandibular condyle and a distally positioned angular process (Fig. 7A). This combination of characters is associated with an increase of the antero-posterior component of the masseter and internal pterygoid muscle forces, thereby implying nearly propalinal mastication (Fig. 7A & B), which is correlated with the decrease of occlusal pressure. It seems that this decrease was compensated for a strong development of the medial layer of the masseter muscle [30] that is inserted in a deep fossa on the dorsomedial side of the enlarged lateral crest (i.e., upper masseteric crest) in rodents of the cavioid type.

Medial movements of the lower jaws are an important component of the power stroke in mammals [39] but Greaves [38] noted that a high mandibular condyle is required to maintain an oblique chewing movement. Indeed, a position of the articular joint above the cuspidate occlusal plane of the molars would change the magnitude of the forces acting on the lever by increasing the medial force components without introducing lateral ones [38]. Conversely, in lying below the occlusal plane, the joint would introduce lateral force components that act against medial movements. In summary, a low mandibular condyle condition implies a decrease of the lateral component of the masseter and internal pterygoid muscles forces that could explain a lateral displacement of the angular process of the mandible. Compared to the ancestral type of rodents [40], all hystricognaths evolved toward a reduction of the height of their mandibular condyle. Vassalo and Verzi [30] suggested that such a lateral displacement of the angular process could have occurred during the evolution of hystricognathous rodents and might be at the origin of the groove (i.e. the gutter enclosed by the alveolus of the incisor and the anterior part of the angular process) and the hystricognathy resulting in a strong latero-medial orientation of mastication.

### The mandible of *Laonastes aenigmamus*, a missing link?

The difficulties in classifying *L. aenigmamus* [10], [11] stem from the fact that it presents a mixture of sciurognathous and hystricognathous characters. The following characters were considered to support hystricognath affinities: the hystricomorphous condition of the skull with an enlarged infraorbital foramen; fusion between the *incus* and *malleus*; the greatly reduced coronoid process; the multiserial microstructure of incisor enamel; the enlarged fourth premolar and the retention of a deciduous fourth premolar; the posteriorly directed penis with S-bend, and comblike bristles projecting forward over the claws [10], [11]. However, most of these characters are non-exclusive to Hystricognathi, they are also found in Ctenodactylidae and should be considered as synapomorphies of Ctenohystrica [6], [10], [41]. The mandible of *L. aenigmamus* also presents a unique combination of characters that partly explains the debates concerning its taxonomic position [10], [11], [14]. Its mandible is characterized by a weak lateral displacement of the angular process and an absence of groove (Fig. 7B). It is clear now that diatomyids acquired independently a *pars reflexa* of the superficial masseter [14]. The large development of the *pars reflexa* was proposed to be at the origin of the formation of the groove and used to define the hystricognathous condition of the jaw [42]. The individualization of a groove is always accompanied by a development of the *pars reflexa* of the superficial masseter in hystricognathous rodents, but *L. aenigmamus* is remarkable because its mandible does not display any groove. Thus, this development of the *pars reflexa* could be only clearly linked to a lateral displacement of the angular process. As such, the groove can be conceived as an achievement toward the lateralization of the angular process, an achievement that diatomyids would have never reached. The contribution from the fossil record was decisive in exploring the morphological variation and understanding evolutionary patterns observed in diatomyids. The occlusal surface of *L. aenigmamus* cheek teeth as well as microwear patterns revealed that diatomyids have developed a strong tendency to propalinal mastication (Fig. 7B) in association with flattening of the tooth crown very early during their evolution, as early as the Oligocene [11], [43], [44]. The development of the groove associated with a lateralization of the angular process and the development of the *pars reflexa* of the superficial masseter is strongly associated to oblique chewing movements (see *Biomechanics of hystricognathous jaw* and Fig. 7). In diatomyids, the acquisition of antero-posterior chewing movements seems to have occurred despite the presence of the *pars reflexa*. We think that such a specialization might explain the lack of groove on their mandible. In fact, the mandible of *L. aenigmamus* exhibits an original combination of morphological characters that can be considered as intermediate between sciurognathous and hystricognathous morphologies.

### Paleontological implications

These results bring new insights into the evolution of hystricognathy and will have profound implications for the interpretation of the fossil record of early hystricognathous rodents. Most of the information available from the fossil material pertains to its morphology and the means to quantify morphological characters have become of great importance. The definition of the hystricognathy is complex and geometric morphometrics seems to be the ideal technique to examine shape variation in the mandibles of rodents. However, in some cases of clear recognition of the hystricognathous condition, the use of morphometrical methods would not be of great interest. For instance, *Tsaganomys altaicus* from the early Oligocene of the Hsanda Gol Formation (Mongolia) is one of the oldest rodents known from a complete skull [45], [46], which have a hystricognathous mandible. Despite the indisputable hystricognathous condition of its mandible, *T. altaicus* retains several plesiomorphic characters that depart from the members of Hystricognathi, such as unfused *malleus* and *incus*, enlarged alisphenoid, and imperforate pterygoid fossa [45]. *T. altaicus* also lacks some of the most diagnostic dental features of the Hystricognathi such as the well-developed hypocone and mesolophule, and the metaloph unconnected to the protocone but usually to the anterior arm of the hypocone [47]. However, *T. altaicus* shares some derived characters with other hystricognathous rodents like multiserial incisor enamel, a reduced lacrimal, and it lacks an internal carotid artery. On the basis on this unique association of features, Bryant and McKenna [45] defined the Hystricognathiformes that comprise all rodents more closely related to the crown group Hystricognathi than to Ctenodactylidae. The definition of the Hystricognathiformes clearly illustrates the problems raised by the typological approach of the morphological variation.

Given the quality of the existing fossil record, using our method of geometric morphometry seems equally conceivable on the extinct forms. The oldest representatives of the clade Hystricognathi are known from the late middle to early late Eocene fossil localities of Africa (« Phiomyidae » [12], [48], [49], [50], [51], [52]), but their origin and early diversification seems to have occurred in Asia [47]. These rodents were mainly described based on dental material, even if mandibular and maxillary remains were recently discovered from an earliest late Eocene locality in the Fayum Depression [52], [53]. From now, the sciurognathous-hystricognathous transition is not documented in the fossil record. Considering their phylogenetic affinities, the fossil record of Diatomyidae is likely to play a pivotal role to illustrate this transition, but their fossil record remains very scarce and characterized by a complete absence of pre-Oligocene representative. Striking convergences have occurred in the evolution of Diatomyidae, and we showed here that their mandible display intermediate morphological features between sciurognathous and hystricognathous jaws. This example, along with the case of Tsaganomyidae or Issiodoromyinae [33], shows that hystricognathy cannot be defined unequivocally in the stem representatives of the suborder. Wood [54], [55] already pointed out such an ambiguity in proposing a North American origin of the Caviomorpha (i.e. South American hystricognaths) from franimorph rodents based on the recognition of an “incipient hystricognathy”. The definition of hystricognathy was even questioned in the present work for some caviomorph rodents (cavioid type, i.e. Hystricognathi) that display, to some extent, a mandible very similar to some sciurognathous members of the order. In that case, their inclusion within the Hystricognathi clade is not based on the condition of the angular process of their mandible (i.e. lateralization) but on other features, notably molecular and some other anatomical details [56], [57], [58], [59], [60]. Our study underlines the interest of using both paleontological and morphometrical analyses applied to well-established morphological and molecular phylogenies to assess morphological evolution.

This study illustrates how a holistic approach allows an objective study of the morphological variation while any typological approach failed because it implied quasi-invariable morphotypes. Our analysis gives the first quantified account of the morphological variation exhibited by the mandibles of rodents. Such a situation, which explains the past difficulty in classifying rodents based on cranial and mandibular characteristics, has the advantage of reflecting the multiple evolutionary paths followed during the evolution of rodents, and unveiled by the quantitative research based upon recent morphological and molecular phylogenies. The key character defining hystricognathy, the lateralization of the angular process of the mandible, was shown to be related to the mechanics of the masticatory apparatus, and especially to oblique masticatory movements. A mechanical model of the muscles and their effect on the movements of mastication will help to verify different hypotheses concerning the link between cranial and dental morphology and the direction of chewing. Moreover, the study of the masticatory biomechanics might yield new insights into the evolution of hypsodont cheek teeth.

The monophyly of extant hystricognaths is well supported morphologically [12], [47], [61], [62], [63], [64]. Hence, one of the most important issues does not actually involve the recognition of the hystricognathous condition of a mandible but more the name used to designate this group of rodents, or more exactly all the extant representatives of this clade. According the mosaic character of evolution, the question is first to determine the order of character acquisition (skull, mandible, etc.) in the many lineages that have arisen, and then, the taxonomical treatment to apply. Did the most basal stem hystricognaths show a hystricognathous condition of their mandible? Probably not, but paradoxically the morphology of their mandible would no more be sufficient to define them as hystricognaths. On one hand, "hystricognathy" as a character could be useless to move forward with phylogenetic analyses; on the other hand the evolution of the Diatomyidae tends to show that we could consider two characters to be independent: the lateral *vs* medial placement of the angular process, and the presence/absence of a ventral groove. From now on, it would be wiser to progress in the quantified description of the morphological variation of the mandible in rodents rather than to propose a partial recognition of the hystricognathous condition. De Queiroz [65] reached the same conclusions with the problematic definition of the class Mammalia that is based on the description of extant groups and not of all basal species likely to have teats. For De Queiroz [65] “*Taxonomists* … *grant more importance to such things as usage, usefulness, and nomenclatural convention priority than to descriptive accuracy*”. We have to acknowledge that the hundred-year-old classification of Tullberg [7] at least addressed the challenge to reconcile molecular studies with morphological data. The enigmatic morphology of the mandible of *Laonastes* may represent one of those cases when morphology is more complex than language.

## Supporting Information

Appendix S1 **List of measured specimens.** *Abbreviations:* MNHN: Museum National d'Histoire Naturelle, Paris. Collection Vertébrés supérieurs Mammifères et Oiseaux; BMNH: Natural History Museum in London; MSUT: Mahasarakham University Herbarium; UMC: Montpellier University Collection.
(DOC)
