# Supplementary material for: Hystricognathy vs Sciurognathy in the Rodent Jaw: A New Morphometric Assessment of Hystricognathy Applied to the Living Fossil Laonastes (Diatomyidae)
Source: PLoS One. 2011 Apr 7;6(4):e18698. doi: 10.1371/journal.pone.0018698 (PMC3072414; doi:10.1371/journal.pone.0018698)
Supplement: Appendix S1 — List of measured specimens. Abbreviations: MNHN: Museum National d'Histoire Naturelle, Paris. Collection Vertébrés supérieurs Mammifères et Oiseaux; BMNH: Natural History Museum in London; MSUT: Mahasarakham University Herbarium; UMC: Montpellier University Collection. (DOC) [file pone.0018698.s001.doc]

**Sciurognathi**

**Ctenodactylidae**

Diet: Grass.

Type of habitat: open areas.

*Ctenodactylus gundi*: BMNH 25.6.3.12, BMNH 25.12.18.7, MNHN CG1975-307

*Ctenodactylus* *vali*: MNHN CG1953-787

*Felovia vae*: BMNH 19.7.7.3226

*Massoutiera mzabi*: BMNH 14.8.21.7, BMNH 12.11.14.55, BMNH 34.8.2.84

**Diatomyidae**

Diet: indet.

Type of habitat: woody areas.

*Laonastes aenigmamus*: MSUT 004003, MSUT 004004, MSUT 004005.

**Hystricognathi**

**Abrocomiidae**

Diet: Grass.

Type of habitat: open areas.

*Abrocoma sp.:* MNHN CG2001-1874

*Abrocoma schistacea:* BMNH 21.6.19.15, BMNH 21.6.19.17

*Abrocoma cinerea:* BMNH 26.1.1.107, BMNH 25.3.1.60

*Abrocoma vaccarum:* BMNH 21.6.24.19

**Capromyidae**

Diet: omnivorous.

Type of habitat: woody areas.

*Geocapromys browinii*: MNHN CG1872-277

*Capromys thoratacus*: BMNH 13176

*Capromys sp.:* MNHN CG1938-850

**Caviidae**

Diet: Grass.

Type of habitat: open areas.

*Cavia porcellus*: BMNH 28.10.11.70, MNHN CG1962-2092

*Cavia aperea*: BMNH 79.210, BMNH 17.05.02.14

*Cavia tschudii*: BMNH 26.5.3.146

*Dolichotis patagonum*: BMNH 2003.339, BMNH 13.11.1.11, BMNH 2003-340, MNHN CG1982-1074, N366

*Galea spixii*: MNHN CG1975-465, MNHN CG1984-660

*Hydrochoerus hydrochaeris*: BMNH 70-2528, BMNH 4.1.9.1, BMNH 3.9.28.1, BMNH 11.10.27.7, MNHN CG1962-2221, MNHN CG1988-197, UMC V71

*Kerodon rupestris*: MNHN CG1982-646

*Microcavia australis*: BMNH 27.5.7.1.114, BMNH 21.7.5.18, BMNH 34.11.4.97, BMNH 34.11.4.100, BMNH 98.3.16.28, BMNH 98.3.16.19

**Chinchillidae**

Diet: Grass.

Type of habitat: open areas.

*Chinchilla lanigera*: BMNH 98.1.8.17, BMNH 98.8.2.9, BMNH 98.8.2.7

*Lagidium peruanum*: MNHN CG1957-1289, MNHN CG1957-1291

*Lagidium viscacia*: BMNH 98.1.3.7, BMNH 20.3.17.69, BMNH 20.3.17.66

*Lagostomus maximus*: BMNH 20.12.22.1, BMNH 9.12.1.42, BMNH 3.6.6.13, MNHN CG1974-336, MNHN CG1974-335

*Lagostomus trichodactylus*: BMNH 96.10.31.1

**Ctenomyidae**

Diet: grass.

Type of habitat: burrowers.

*Ctenomys budini*: BMNH 21.11.1.86

*Ctenomys coludo*: BMNH 34.11.4.152, BMNH 34.11.4.159, BMNH 34.11.4.151

*Ctenomys haigi*: BMNH 19.1.5.19

*Ctenomys knighti*: BMNH 20.3.17.58

*Ctenomys opimus*: MNHN CG1961-671

**Cuniculidae**

Diet: fruit-leaf.

Type of habitat: woody areas.

*Cuniculus paca*: BMNH 76-684, BMNH 76-686, BMNH 2007-265, BMNH 52.383, MNHN CG1937-1241, MNHN CG1997-646

*Cuniculus taczanovski*: BMNH 34.9.10.187, BMNH 5.3.5.9, BMNH 5.7.5.14

**Dasyproctidae**

Diet: fruit-leaf.

Type of habitat: woody areas.

*Dasyprocta azarae*: BMNH 76.696, CG1885-855

*Dasyprocta fuliginosa*: BMNH 77.93

*Dasyprocta leporina*: MNHN CG1958-726, BMNH 1930-2263

*Dasyprocta punctata*: BMNH 51.2.17.87, CG1932-2891

*Dasyprocta sp.*: BMNH 10.9.29.23

*Dasyprocta variegata*: MNHN CG1932-2890, MNHN CG1932-2891

*Myoprocta acouchy*: MNHN CG1936-1442, MNHN CG1999-1081, MNHN CG1998-2259, MNHN CG1998-2258, BMNH 3466, BMNH 27.8.11.52, UMC V1187

*Myoprocta pratti*: BMNH 34.9.10.192, BMNH 34.9.10.193

**Dinomyidae**

Diet: fruit-leaf.

Type of habitat: woody areas.

*Dinomys branicki*: MNHN CG1990-658, BMNH 34.9.10.191, BMNH 12.15.4

**Echimyidae**

Diet: fruit-seed.

Type of habitat: woody areas.

*Echimys chrysurus*: UMC V-1636, MNHN CG1999-1046, MNHN CG1999-1082

*Echimys semivillosus*: BMNH 98.12.1.19

*Isothrix sinnamariensis*: UMC V-1585, UMC V-1708

*Makalata didelphoïdes*: BMNH 97.6.7.50, CG1995-213, UMC V-1566, UMC V-1251

*Mesomys hispidus*: UMC V-1047, UMC V-1049, BMNH 24.2.4.15, MNHN CG2003-771, MNHN CG2001-2233, BMNH 26.5.3.133

*Myocastor coypus*: BMNH 61-656, BMNH 1981-1428, MNHN CG1992-672, MNHN CG1935-887

*Proechimys cayenensis*: MNHN CG1938-2928, MNHN CG1998-682

*Proechimys cuvieri*: UMC V-1174, MNHN CG1998-1824, MNHN CG1998-696, MNHN CG1997-655, MNHN CG1996-653, MNHN CG1997-660, MNHN CG1996-663, MNHN CG1997-649, MNHN CG1997-659, MNHN CG1997-664, MNHN CG1997-651.

*Proechimys oris*: MNHN CG2006-462, MNHN CG2006-323, MNHN CG2006-324, MNHN CG2006-315

*Trichomys aperoides*: CG1983-896

**Erethizontidae**

Diet: fruit-leaf.

Type of habitat: woody areas.

*Atherurus africanus*: BMNH 48.1291, MNHN CG2003-57

*Atherurus macrourus*: BMNH 33.4.1.490, BMNH 33.4.1.484, BMNH 55.3206

*Chaetomys subpinosus*: BMNH 3.9.4.86

*Coendou bicolor*: BMNH 34.9.10.186

*Coendou qouiy*: BMNH 25.5.1.10

*Coendou vestitus*: MNHN CG1929-632

*Erethizon dorsatum*: BMNH 36.11.6.28, BMNH 36.11.6.27, BMNH 36.11.6.26

*Shiggurus mexicanus*: MNHN CG1916-81, MNHN CG1916-79

**Hystricidae**

Diet: omnivorous.

Type of habitat: open areas.

*Hystrix cristata*: BMNH 62.461

*Hystrix africae*: BMNH 2.12.1.22

*Hystrix indica*: BMNH 77.407

*Hystrix somaticus*: BMNH 6.5.4.9

Diet: omnivorous.

Type of habitat: woody areas.

*Thecurus crassispinus*: BMNH 92.10.1.5, BMNH 40.626, BMNH 40.625

*Thecurus pumilus*: BMNH 94.12.1.15

*Thecurus sumatrae*: BMNH 40.624

*Trichys lipura*: BMNH 71.3061, BMNH 71.3060, BMNH 89.1.8.7

**Octodontidae**

Diet: grass.

Type of habitat: open areas.

*Octodon degus*: BMNH 9.6.13.4, BMNH 4.1.7.17, MNHN CG2001-1884, MNHN CG2001-1883

*Octodontomys gliroides*: BMNH 21.11.1.76

Diet: roots.

Type of habitat: burrowers.

*Spalacopus sp.*: MNHN CG2001-1867, MNHN CG2001-1867

**Petromuridae**

Diet: fruit-seed.

Type of habitat: open areas.

*Petromys typicus*: BMNH 25.1.2.217, BMNH 4.2.2.102, BMNH 23.5.9.170

**Thryonomyidae.**

Diet: Grass.

Type of habitat: open areas.

*Thryonomys swinderianus*: BMNH 26.11.24.63, BMNH 76.195, BMNH 1991.178, BMNH 73.1775, MNHN CG2003-65
